# The Effect of Cumin on the Formation of *β*-Carboline Heterocyclic Amines in Smoked Meat and Simulated Systems

**DOI:** 10.3390/foods14020299

**Published:** 2025-01-17

**Authors:** Xiuxiu Liu, Wenyu Chen, Minghao Sun, Xufang Lv, Xing Shen, Zhongping Chai, Maomao Zeng

**Affiliations:** 1 College of Resources and Environment, Xinjiang Agricultural University, Urumqi 830052, China; liuxiuxiu3629@163.com (X.L.); chenwenyu666666@163.com (W.C.); sun18453303381@126.com (M.S.); 15035048162@163.com (X.L.); 2 Xinjiang Key Laboratory of Soil and Plant Ecological Processes, Xinjiang Agricultural University, Urumqi 830052, China; 3 State Key Laboratory of Food Science and Resources, Jiangnan University, Wuxi 214122, China; mmzeng@jiangnan.edu.cn; 4 School of Food Science and Technology, Jiangnan University, Wuxi 214122, China

**Keywords:** cumin, smoked meat, *β*-carboline heterocyclic amines

## Abstract

In this study, we aimed to investigate the inhibitory effects of cumin and cumin extracts from different origins (Hami, Turpan, and Hetian) on the formation of *β*-carboline heterocyclic amines (HCAs) in smoked meat and simulated systems, and to assess their potential as natural inhibitors in the food industry. The novelty of our research lies in the comprehensive comparative analysis of cumin extracts from different origins, which has not been fully explored in previous studies. We first conducted a quantitative analysis of the total phenol and flavonoid content in cumin extracts from the three origins and evaluated their antioxidant capacities. Subsequently, through simulation experiments, we assessed the inhibitory effects of these extracts on the formation of *β*-carboline heterocyclic amines and determined their free radical scavenging abilities. To further validate the practical application potential of these extracts, we prepared meat patty samples containing different concentrations of cumin powder, simulating actual processing conditions. The experimental results showed that while the total phenol content in cumin extracts from all origins was similar, averaging around 1.56 mg/g, there was a significant difference in the total flavonoid content, with the highest level observed in the Hetian cumin extract at 6.7 mg/g. Additionally, the Hetian cumin extract demonstrated superior antioxidant capacity, with an FRAP antioxidant activity reaching 21.04 μM TE/g dw, the highest among all samples. Our study also found that the inhibitory effect of cumin extracts on HCA formation was closely related to their free radical scavenging ability, with the Hetian cumin extract showing the strongest scavenging capacity. The addition of cumin powder to meat patties significantly reduced the content of *β*-carboline heterocyclic amines, particularly at lower cumin concentrations. In summary, our research results highlight the potential of cumin, especially from Hetian, as a natural inhibitor of *β*-carboline heterocyclic amine formation in processed meats. This study not only provides the food industry with a potential natural additive to improve food safety and quality, but also offers new directions for future research, namely by comparing natural plant extracts from different origins to explore their potential applications in food processing.

## 1. Introduction

Cumin is one of the oldest seeds and is the second most popular spice after black pepper [1]. Its unique flavor and seasoning properties make it commonly used as a flavoring agent in herbal beverages and homemade cuisines [2], rendering the study of its components highly significant. Cumin is rich in nutrients and bioactive substances such as proteins and fats, along with polyphenols and flavonoids. It possesses the ability to scavenge, reduce oxidative stress, and enhance immunity, among other health benefits. Cumin is also widely applied in various fields such as food and cosmetics. Polyphenols and flavonoids in cumin exhibit antioxidant properties [3], which aid the human body in eliminating free radicals and mitigating oxidative stress. Research by Ding et al. [4] demonstrated that polyphenols such as chlorogenic acid, epicatechin, and quercetin can inhibit the formation of Harman and Norharman in charcoal-grilled mutton. Jing et al. [5] found that flavonoids and flavonoid components like rutin, hesperidin, and flavanone in purslane could simultaneously inhibit the formation of PhIP, TMIP, Norharman, and AαC in grilled beef patties, with rutin showing the most significant inhibitory effect in a concentration-dependent manner. Dong et al. (2024) [6] found the structure–activity relationship and influence mechanism of polyphenols on the formation of heterocyclic aromatic amines (HAA) in hot processed foods. Heterocyclic aromatic amines are strong carcinogens mainly produced in hot processed foods. Natural polyphenols can effectively control the formation of HAAs in hot processed foods and reduce their harm to human health. Numerous studies have revealed that the addition of natural antioxidants such as polyphenols, flavonoids, and vitamins can significantly inhibit HCA formation.

1-methyl-9h-pyridine [4,3-b] indole (Harman) and 9H-pyridine [4,3-b] indole (Norharman) are two main *β*-carboline heterocyclic amines (HCAs) that are formed during hot processing of foods, especially in protein-rich cooked meat products. These compounds are carcinogenic and mutagenic, so knowing their levels in food is critical to assessing food safety. Beta-carboline HCAs are a class of compounds formed in this context, especially in cooked meat products. Harman and Norharman are found as *β*-carboline alkaloids in nature [7]; they are widely distributed in nature and primarily synthesized endogenously by plants [8]. These *β*-carboline alkaloids play a pivotal role in herbal remedies, demonstrating a spectrum of therapeutic effects, including antioxidant, antiviral, antibacterial, and anti-inflammatory capabilities [9]. However, Harman and Norharman are known to exert considerable adverse health impacts, notably their potential to induce neurological damage and to exacerbate the effects of additional cancer-causing agents [10]. The formation mechanism of *β*-carboline HCAs is complex, involving the oxidation of tryptophan and a series of chemical reactions during heating, ultimately leading to the generation of these toxic compounds [11]. *β*-Carboline HCAs are bioactive and toxic compounds widely present in foods, and efforts should be made to reduce their production and consumption in our daily diets.

Cumin, with its rich nutritional value and health benefits, is a preferred seasoning in our daily diets, whereas *β*-carboline HCAs should be minimized in our diets due to their potential health risks. Previous studies have shown the effect of cumin on HCA formation in smoked meat. Therefore, this study primarily investigated the inhibitory effect of bioactive substances in cumin extracts on *β*-carboline HCA formation in smoked meat and confirmed that cumin extracts from different origins could inhibit HCAs by scavenging free radicals within them. Finally, the inhibitory effect of cumin on HCAs was verified by adding cumin powder from different origins and at different concentrations to simulated meat patty systems.

## 2. Materials and Methods

### 2.1. Experimental Materials

The experimental materials were mature cumin seeds produced in Xinjiang (Hami, Turpan, and Hetian), China. After impurity removal, drying, and grinding, the cumin powder was stored at −20 °C and retrieved for use as needed. 4-Oxo-TEMPO (purity ≥ 95.0%) was purchased from Shanghai Aladdin Biochemical Technology Co., Ltd. (Shanghai, China) Gallic acid and rutin standards (purity ≥ 96%) were obtained from Shanghai Macklin Biochemical Co., Ltd. Folin–Ciocalteu reagent, 2,2-azinobis (3-ethylbenzothiazoline-6-sulfonic acid) (ABTS), 2,4,6-tri(2-pyridyl)-s-triazine (TPTZ), and 6-hydroxy-2,5,7,8-tetramethylchroman-2-carboxylic acid (Trolox) were purchased from Sigma-Aldrich, St. Louis, MO, USA. Other solvents and reagents were obtained from Sinopharm Chemical Reagent Co., Ltd., Shanghai, China. All reagents and solvents were of analytical grade.

### 2.2. Instruments

The SCC61 E self-cooking center, manufactured by Rational AG of Germany; the Acquity UPLC TQD ultra-performance liquid chromatography tandem triple quadrupole mass spectrometry (UPLC-MS/MS) system, along with the Waters Acquity UPLC BEH C18 column (2.1 mm × 100 mm, 1.7 μm), both from Waters Corporation, Milford, MA, USA; the SpectraMax 190 microplate reader from Molecular Devices, San Jose, CA, USA; the SB-4200DTD ultrasonic cleaner from Ningbo Xinzhi Biotechnology Co., Ltd., Ningbo, China; the Waters e2695 high-performance liquid chromatography instrument (HPLC-PDA) equipped with a 2998 photodiode array detector (PDA), also from Waters Corporation, USA; the EMXplus-10/12 electron spin resonance spectrometer from Bruker, Germany; and the Seven Easy pH meter from Mettler Toledo, Greifensee, Switzerland, were utilized in this study. AX205 (Mettler Toledo, Switzerland): Eppendorf 5425 (Eppendorf, Hamburg, Germany), LGJ-25C freeze dryer (Beijing Sihuan Qihang Technology Co., LTD., Beijing, China), AH-30 automatic homogenizer, Fotectorr-04HT high flux phase extractor, AutoEva-60 automatic nitrogen concentrator, and Mem Spring nitrogen generator were also utilized (Xiamen Ruike Instrument Co., Ltd., Beijing, China).

### 2.3. Preparation of Cumin Ethanol Extract

The cumin powder ethanol extract was prepared according to the method described by Yeoh and Ali [12] with slight modifications. Briefly, 2 g of cumin powder was added to 40 mL of 70% ethanol solution, and ultrasonic-assisted extraction was performed three times (30 min each, 30 °C). The combined extracts were centrifuged (4 °C, 4000 rpm, 10 min), and the supernatant was lyophilization. The lyophilization powder was dissolved in 4 mL of methanol and stored at −20 °C for further use.

### 2.4. Establishment of β-Carboline HCA-Simulated System

In this study, a *β*-carboline HCA-simulated system (Table 1) was adopted as the basis. Additionally, cumin extracts at different concentrations were added to investigate their impact on the simulated system. The selection of these concentrations was based on relevant literature and experimental experience, and was fully validated and optimized prior to the experiments.

### 2.5. Determination of Total Phenolic and Total Flavonoid Contents

The total phenolic content was determined using the Folin–Ciocalteu method described by Singleton and Rossi [13] with appropriate modifications. First, a gallic acid standard solution ranging from 0 to 0.24 mg/mL was prepared. One mL of a 10-fold diluted Folin–Ciocalteu reagent was added to 0.25 mL of the standard solution, followed by 3 mL of sodium carbonate solution (75 g/L). The mixture was diluted to 10 mL with distilled water and allowed to react in the dark for 2 h. The standard curve was obtained by measuring the A765 value. Distilled water was used as the blank control, and cumin extracts from different origins were substituted for the standard solution. The total phenolic content was calculated using the standard curve and expressed as mg GAE/g cumin powder (dw).

The total flavonoid content was determined using the aluminum chloride colorimetric method described by Fu et al. [14] with slight modifications. First, a 0.5 mg/mL rutin standard solution was prepared in 70% ethanol. From 0 to 1.0 mL of the standard solution was taken and diluted to 4 mL with distilled water. Then, 0.3 mL of 5% sodium nitrite solution (allowed to stand for 5 min), 0.3 mL of 10% aluminum chloride solution (allowed to stand for 6 min), and 2 mL of 1 mol/L sodium hydroxide solution were added sequentially. The mixture was diluted to 10 mL and allowed to develop color for 10 min. The A510 value was measured to plot the standard curve. An appropriate amount of cumin extract from different origins was substituted for the standard solution, and the total flavonoid content was obtained from the standard curve and expressed as mg RE/g cumin (dw).

### 2.6. Determination of Antioxidant Capacity

The ABTS radical stock solution and working solution were prepared according to the methods described by Quan et al. [15] and Garzón et al. [16] with slight modifications. First, a mixture of ABTS (7 mmol/L) and potassium persulfate (2.45 mmol/L) in ultrapure water was prepared and allowed to stand in the dark at room temperature for 12–16 h. Before use, the ABTS radical stock solution was diluted with phosphate-buffered solution (pH 7.4, 0.2 mol/L PBS) to an A734 value of 0.700 (±0.020) to prepare the ABTS working solution. Trolox standard solutions ranging from 0 to 1000 μmol/L were prepared, and measurements were performed using a 96-well plate. First, 10 μL of the standard solution was added to each well, followed by 190 μL of the ABTS working solution. The mixture was allowed to react in the dark at room temperature for 10 min. The A734 value was measured, with PBS used as the blank control to obtain the standard curve. After substituting the standard solution with the sample solution, the ABTS radical scavenging capacity of cumin (dw) was obtained in μM/g.

The FRAP working solution was freshly prepared according to the method described by Qie et al. [17] with slight modifications. The working solution was obtained by mixing TPTZ stock solution (10 mmol/L in 40 mmol/L HCl), FeCl3 solution (20 mmol/L), and acetate buffer (0.3 mol/L, pH 3.6) in a ratio of 1:1:10 (*v*/*v*/*v*) and allowing it to stand at 37 °C for 1 h. Trolox standard solutions ranging from 0 to 1000 μmol/L were prepared, and 10 μL of the standard solution and 190 μL of the FRAP working solution were added to each well of a 96-well plate for reaction at room temperature for 30 min. Distilled water was used as the blank control to obtain the standard curve. Finally, the sample solution was substituted for the standard solution, and the FRAP value of the sample was obtained and expressed as μM/g cumin (dw).

### 2.7. Determination of Free Radicals in the Simulated System

The total radical content was determined by referring to the method described by Wang et al. [18]. Before heat-treating the *β*-carboline HCA-simulated system, 200 μL of the spin trap agent 4-Oxo-TEMPO (100 mmol/L) was added to each sample and mixed well. After the reaction, a capillary glass tube with an inner diameter of 1.5 mm was used to aspirate the reaction solution to a depth of 1 cm in the tube, which was then sealed with Vaseline. The capillary glass tube was placed at the bottom of an NMR tube, which was subsequently placed in the resonator cavity of the electron spin resonance spectrometer. After waiting for the resonator to tune and balance, the total spin count of the sample was measured. The parameters were as follows (Table 2): center field, 3360 G; sweep width, 100 G; sweep time, 30 s; sweep count, 3; microwave power, 20 mW; modulation amplitude, 1.0 G.

### 2.8. Determination of β-Carboline HCAs in Smoked Meat Patties

#### 2.8.1. Preparation of the Sample

Following the protocol detailed by Chen et al. [19], the liberation and enrichment of free and protein-binding heterocyclic amines (HCAs) from smoked meat samples were executed. The preparation steps are as follows. Add 3 g of lyophilization smoked meat powder to 30 mL of 1 mol/L sodium hydroxide solution, and homogenize using an automatic homogenizer at 12,000 rpm for 1 min. Subsequently, incorporate 13 g of diatomaceous earth and 50 mL of ethyl acetate into the homogenate and mix thoroughly. Then, subject the mixture to ultrasonic treatment using an ultrasonic cleaner at 40 kHz for 30 min. Afterwards, centrifuge at 10,000× *g* for 10 min to collect the supernatant. Repeat the extraction process twice, and then concentrate the solution to less than 10 mL using nitrogen for solid-phase extraction.

Beyond the supernatant, the sediment post-centrifugation was also harvested. To a 48 mL robust, pressure-tolerant vial, 40 mL of a 6 M hydrochloric acid solution was introduced. After a nitrogen purge for 60 s to eliminate oxygen, the vial was subjected to a thermal treatment at 110 °C for a full day. Upon completion of the heating cycle, the vial was swiftly cooled, and the supernatant was separated post-centrifugation. This supernatant was then diluted to a total volume of 250 mL with ultrapure water, from which a 10 mL portion was allocated for solid-phase extraction.

#### 2.8.2. Solid-Phase Extraction

Solid-phase extraction was performed using a high-throughput automated solid-phase extraction system. Different pretreatment methods were applied based on the properties of free and protein-binding HCAs. For free HCAs, the Oasis MCX column was activated with 6 mL of water, 6 mL of methanol, and 6 mL of ethyl acetate. For protein-bound HCAs, the Oasis MCX column was activated with 6 mL of methanol, 6 mL of water, and 6 mL of 0.1 mol/L HCl.

Upon the introduction of the purification-targeted sample extract into the solid-phase extraction cartridge, a series of washes were conducted to eliminate impurities. Initially, the column was treated with 6 mL of 0.1 mol/L HCl, followed by a 6 mL methanol rinse, aligning with the methodology reported by Chen et al. [19]. Finally, the retained heterocyclic amines were eluted using 6 mL of a methanol-ammonium hydroxide mixture (19:1, *v*/*v*). The eluate was then dried under nitrogen at room temperature and reconstituted in 300 μL of methanol. Lastly, the solution was prepared for liquid chromatography-mass spectrometry (LC-MS) injection and analysis.

#### 2.8.3. Determination of *β*-Carboline HCAs

The quantification of heterocyclic amines (HCAs) derived from smoked meat was accomplished through ultra-performance liquid chromatography-tandem mass spectrometry (UPLC-MS/MS). The chromatographic resolution of HCAs was facilitated by an Acquity BEH C18 column (dimensions: 2.1 × 100 mm, particle size: 1.6 μm, manufactured by Waters Corporation, USA), which was maintained at a temperature of 45 °C throughout the process. The mobile phase was a mixture of pure acetonitrile and a 0.1% formic acid solution, following a predefined gradient elution scheme: from 0 to 2 min with 2% solvent A; from 2 to 12 min with a linear increase to 20% A; from 12 to 14 min with 100% A; from 14 to 17 min reverting to 2% A [20]; and from 17 to 20 min maintaining 2% A. The column’s flow rate was maintained at 0.3 mL per minute, with an injection volume of 2 μL for each sample. In the mass spectrometry phase, a multiple reaction monitoring (MRM) approach was adopted under positive ion detection conditions. The parameters were optimized as follows: the temperature of the ion source was 100 °C, the desolvation temperature reached 400 °C, and the desolvation gas flow was set at 700 L/h. The capillary voltage was adjusted to 3.5 kV, with nitrogen serving as the cone gas at a flow rate of 50 L/h, and argon as the collision gas at a flow rate of 0.15 mL/min. The mass range for scanning was set between 2 and 2000 Da [21].

### 2.9. Determination of Final β-Carboline HCAs in the Simulated System

Employing the procedure detailed by Barzegar et al. [22], we conducted the isolation and quantification of *β*-carboline heterocyclic amines (HCAs). Initially, 2 mL of the post-centrifugation reaction suspension was aliquoted, followed by the introduction of 100 µL of a 2 M NaOH solution. Subsequent extractions were carried out using 5 mL portions of ethyl acetate, repeated thrice, with the collected fractions pooled together. This pooled extract was subjected to nitrogen evaporation at 50 °C and then reconstituted in 300 µL of methanol. After a 10 min centrifugation, the supernatant layer was decanted into a chromatography vial in preparation for analysis. The HCA content was quantified utilizing an Acquity UPLC-MS/MS setup. The separation was achieved on an Acquity UPLC BEH C_18_ column (2.1 mm × 100 mm, 1.7 μm), maintained at 45 °C, with the sample introduction area cooled to 4 °C. The mobile phase was a binary mixture of solvent A: acetonitrile and solvent B: a 3 mM ammonium acetate solution adjusted to pH 4.75, delivered at a flow rate of 0.3 mL/min. A gradient elution protocol was applied with the following timeline and solvent A percentages: 2 min at 2%, increasing to 20% by 12 min, full transition to 100% by 14 min, returning to 2% by 17 min, and maintaining at 2% through 20 min. The sample injection volume was 2 μL. In the mass spectrometry phase, an electrospray ionization (ESI) source in positive mode was utilized for ionization, with multiple reaction monitoring (MRM) for data acquisition. The ESI+ settings included a desolvation gas temperature of 400 °C, an ion source temperature of 110 °C, a capillary voltage of 3500 V, a cone gas flow of nitrogen at 50 L/h, and a collision gas flow of argon at 0.13 mL/min.

### 2.10. Statistical Analysis

The quantitative outcomes derived from UPLC-MS/MS technology were evaluated utilizing the MassLynx V4.1 software, provided by Waters Corporation in the United States. To analyze the dataset, a one-way ANOVA with a completely randomized design was implemented with the aid of Statistix 9.0 software, originating from Tallahassee, FL, USA. Subsequent to the ANOVA, Tukey’s HSD test was applied for multiple comparisons at a significance level of *p* = 0.05. The results were depicted as the mean ± standard deviation, with each set of data being measured three times (n = 3). For the PCA analysis of the heterocyclic amines, SIMCA 14.1 software from Umetrics in Umea, Sweden, was utilized. The assessment of Spearman’s rank correlation was conducted with the Origin Pro 2021b software, a product of OriginLab Corporation based in Northampton, MA, USA. For visual representation, graph plotting was carried out using both Origin Pro 2021b and Anaconda Jupyter Notebook V6.4.8 software.

## 3. Results

### 3.1. Analysis of Total Phenolic and Total Flavonoid Contents in Cumin Extracts

Numerous studies have confirmed that polyphenolic and flavonoid compounds exhibit significant inhibitory effects on HCAs. Oguri et al. [23] investigated the inhibitory effects of flavonoid compounds such as EGCG and quercetin on HCAs in simulated systems and found that their inhibition rates reached up to 80%. Oz and Kaya [24] discovered that adding 1% red pepper to grilled beef patties reduced the total HCAs content by 94%, likely due to the flavonoid antioxidants such as quercetin and luteolin present in red pepper. As a common seasoning for meat products, cumin is rich in nutrients and antioxidants, contributing to its unique flavor and nutritional benefits. In this study, we investigated the total phenolic and total flavonoid contents in cumin extracts from different origins (Hami, Turpan, and Hetian). The results indicated that there were no significant differences in total phenolic content among the cumin extracts from the three origins, but there were significant differences in total flavonoid content (Table 3). Total phenolic content is one of the indicators for measuring the antioxidant capacity of plants, and cumin from Hetian had a slightly higher total phenolic content than that from Hami and Turpan. Additionally, the total flavonoid content was highest in the Hetian group at 6.7 mg/g, significantly higher than that in the Hami group (*p* < 0.05), while the difference with the Turpan group was not significant (*p* > 0.05). However, all values were higher than the total flavonoid content of 2.35 mg/g reported by Rebey et al. [25], than all our measured values, indicating that the cumin varieties we studied may offer superior antioxidant benefits. These results underscore the importance of selecting cumin with higher flavonoid content for applications where reducing HCAs is a priority, such as in the preparation of grilled meats.

### 3.2. Antioxidant Capacity of Cumin Extracts

The in vitro antioxidant capacities of cumin extracts from different origins were analyzed (Table 4). Based on the ABTS and FRAP assays, the Hetian group exhibited slightly better antioxidant capacity than the cumin from the other two origins. Specifically, the Hetian group had the highest FRAP antioxidant activity at 21.04 μM TE/g dw, while the Hami group had the lowest at 17.2 μM TE/g dw. The ABTS antioxidant activities of the three origins were very close. Although the results were similar, subtle differences were observed, with the Hetian group showing slightly higher values than the other two groups. Based on these results, it can be inferred that cumin from Hetian has stronger in vitro antioxidant capacity.

### 3.3. Effect of Cumin Extracts on the Formation of β-Carboline HCAs

**Figure 1:**
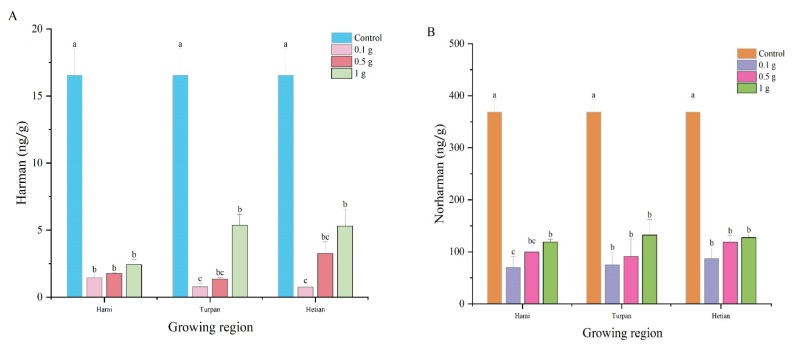
The effect of cumin extract on the formation of *β*-Carbolines HAs in a *β*-Carbolines HAs model system. (**A**) Harman, (**B**) Norharman. Different lowercase letters in the same column indicate significant differences (*p* < 0.05).

Figure 1A explores the effect of cumin extract additions from different origins (Hami, Turpan, and Hetian) on Harman content. The experiment included a control group and three treatment groups with cumin extract additions of 0 g, 0.1 g, 0.5 g, and 1 g of lyophilization powder. The control group had a Harman content of 16.56 ng/g. In the treatment groups with cumin extract additions, Harman content increased significantly with increasing amounts of cumin extract. At 0.1 g, 0.5 g, and 1 g additions, the Harman content in all origin groups was significantly lower than that in the control group. Additionally, even at the same cumin extract addition, there were differences in Harman content among the different origin groups. Specifically, when 0.1 g of cumin extract was added, the Harman content in the Hami group was 1.44 ng/g, while that in the Hetian and Turpan groups was 0.75 ng/g and 0.78 ng/g, respectively, representing reductions of 47.91% and 45.83% compared to the Hami group. These results suggest that the addition of cumin extract not only reduces Harman content, but also that its antioxidant capacity may be related to the decrease in Harman content, as antioxidants can inhibit the formation of heterocyclic amines during hot processing.

Figure 1B shows the changes in Norharman content in the simulated system with different cumin extract additions (0 g, 0.1 g, 0.5 g, 1 g) from different origins (Hami, Turpan, and Hetian). The results revealed significant differences in Norharman content among the different cumin extract additions and origins. The control group had significantly higher Norharman content than the treatment groups with cumin extract additions, indicating that cumin extracts from different origins and at different additions all inhibited Norharman formation. In the treatment groups, Norharman content increased with increasing cumin extract addition. When 0.1 g of cumin extract was added, the Norharman content in the Hetian and Turpan groups was 87.44 ng/g and 74.82 ng/g, respectively, representing increases of 24.9% and 6.9% compared to the Hami group (69.96 ng/g). At 1 g of cumin extract addition, the Norharman content in the Turpan group was significantly higher than that in the Hami and Hetian groups, increasing by 11.6% and 4.4%, respectively. These results indicate that the response of Norharman content to cumin extract addition varied among different origin groups, Additionally, this response may be related to the antioxidant capacity of cumin extract, because antioxidant capacity can affect the formation and accumulation of heterocyclic amines.

### 3.4. Free Radical Scavenging Ability of Cumin Extracts

**Figure 2:**
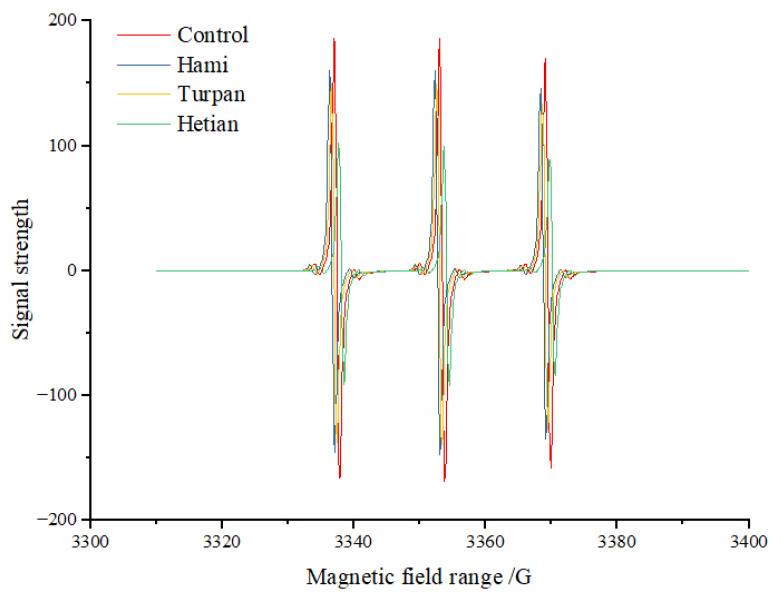
The scavenging ability of free radicals in the *β*-Carbolines HAs-aldehyde mixture system by cumin extract.

Reducing sugars and amino acids can generate free radicals through oxidative reactions at high temperatures. Alkyl radicals and pyrazine radicals have been confirmed to participate in the formation of quinoxaline HCAs [26]. Currently, it is generally believed that *β*-carboline HCAs are primarily formed through the pyrolysis of proteins or amino acids at high temperatures. Tryptophan undergoes Amadori rearrangement followed by dehydration, and the resulting dehydration product forms a conjugated oxonium ion through a *β*-elimination reaction under the action of epoxy lone pair electrons. The oxonium ion then undergoes dehydration and conjugate extension to maintain a stable structure, followed by intramolecular nucleophilic substitution to form *β*-carboline HCAs. However, Xu et al. [27] found that free radicals may also play an important role in the formation of *β*-carbolines. Therefore, electron spin resonance (ESR) technology was used to investigate the changes in free radicals in the *β*-carboline HCA-simulated system with the addition of cumin extract lyophilization powder (0.1 g) (Figure 2). The results showed that the signal intensity (amplitude) in the control group was higher than that in the samples with cumin extracts from Hami, Turpan, and Hetian, indicating that the free radical content in the control group was significantly higher than that in the experimental groups with cumin extract additions. Additionally, the characteristic components of cumin extracts from different origins had significant effects on free radical scavenging ability. Differences in growth environment, soil, climate, and other factors may lead to variations in the composition of cumin extracts, thereby affecting their antioxidant activities and free radical scavenging abilities. As shown in the figure, the scavenging ability of cumin extract from Hetian was the strongest, while that from Hami was the weakest.

The higher the free radical content, the greater the total spin count. The total spin count in the control group was11.85*1015, while those in the samples with cumin extracts from Hami, Turpan, and Hetian were10.01 × 1015, 7.36 × 1015 and 6.94 × 1015, respectively, all significantly lower than that in the control group. In particular, the total spin count in the sample with cumin extract from Hetian was reduced by 41.43% compared to the control group, representing a significant decrease. This is consistent with the significant effect of Angelica extract in reducing the production of heterocyclic amines, which is consistent with our observed reduction in total spin number of cumin extract, further confirming the potential of natural plant extracts in inhibiting free radical and heterocyclic amines production [28].The inhibitory effect of cumin extracts on *β*-carboline HCA formation may be related to the free radical scavenging ability of their polyphenolic and flavonoid components. This finding is consistent with the results of Shao et al. [29], who evaluated the free radical scavenging abilities of nine flavonoid compounds in a glucose–creatinine–glycine-simulated system and found that proanthocyanidins and quercetin had strong free radical scavenging abilities and strong inhibitory effects on HCA formation. Many studies have shown that grape seed extract [29], avocado peel extract [30], rose tea extract [31], and other extracts can inhibit HCA formation by scavenging free radicals. Khan et al. [32] also found a strong correlation between the free radical scavenging ability of perilla nut extract and its inhibitory effect on HCAs in chicken breast meat.

### 3.5. Effect of Cumin Powder on the Formation of β-Carboline HCAs in Smoked Meat Patties

**Figure 3:**
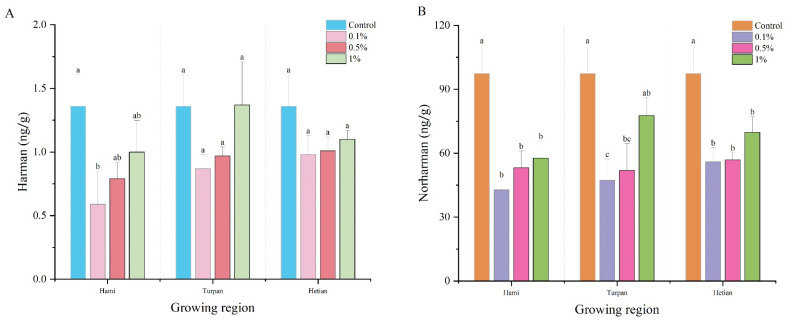
Effects of cumin powder of Hami, Turpan and Hotan at different concentrations on the content of *β*-carbine heterocyclic amine (HAs) in smoked meat patties: (**A**) Harman; (**B**) Norharman. Different lowercase letters in the same column indicate significant differences (*p* < 0.05).

Figure 3A,B show the changes in Harman and Norharman content in smoked meat patties after the addition of cumin powder from different origins (Hami, Turpan, and Hetian) at different concentrations (0, 0.1%, 0.5%, and 1%) and smoking at 70 °C for 3 h. For Harman content, in most cases, the Harman content in the meat patties with cumin powder additions was significantly lower than that in the patties without cumin powder, indicating that cumin powder had a certain inhibitory effect on Harman formation. However, when the addition amount increased to a certain level, the Harman content gradually increased. Specifically, the Harman content in the meat patties with 1% Turpan cumin powder was the highest at 1.37 ng/g, slightly higher than that in the patties without cumin powder (1.36 ng/g). For cumin powder from different origins, there were differences in Harman content in the meat patties with cumin powder additions. When the addition amount was 0.1%, the Harman content in the Hetian group was the highest, being 66.1% and 12.64% higher than that in the Hami and Turpan groups, respectively.

The average Norharman content in the patties without cumin powder was 97.43 ng/g, which is significantly higher than that in the patties with cumin powder additions from different origins. This indicates that the addition of cumin powder effectively reduced Norharman formation. Additionally, Norharman content increased with increasing additions of cumin powder. Similar effects were observed with cumin powder from the three origins. Among them, the addition of Turpan cumin powder had the most significant effect on Norharman content in the meat patties, increasing from 47.3 ng/g at 0.1% addition to 77.7 ng/g at 1% addition, representing an increase of 64.27%. However, compared to the simulated system in Section 3.3, the inhibitory effect of cumin powder on Harman and Norharman in actual meat patties was significantly weaker. This may be due to the complex matrix of the meat patty system, with interactions among various food components such as lipids and proteins, which reduced the inhibitory effect [33]. Deng et al. [34] found that the combination of some amino acids and EGCG and other polyphenols can significantly reduce the formation of PhIP, similar to the results of this study. Notably, cumin powder had a better inhibitory effect on *β*-carboline HCAs at lower addition amounts, consistent with previous findings. Based on these results, subsequent experiments should focus on exploring the inhibitory mechanism of cumin powder at lower additions [35]. Based on these results, future studies should focus on exploring the inhibition mechanism of cumin powder at low supplemental levels.

## 4. Conclusions

In conclusion, our study comprehensively evaluated the inhibitory effects of cumin powder on the formation of *β*-carboline heterocyclic amines (HCAs) in smoked meat systems, aligning with our initial research objectives. We found that the addition of cumin powder significantly reduced the formation of *β*-carboline HCAs, with a notable decrease in Harman content in meat patties containing cumin compared to those without. However, an increase in cumin addition beyond a certain threshold led to a resurgence in Harman content, while Norharman production remained consistently inhibited. Cumin from Hetian, with its superior bioactive compound content and antioxidant capacity, demonstrated the most effective inhibition of HCAs, particularly at lower levels of addition. The correlation between the inhibition effect and the concentration and geographical origin of cumin extracts suggests that these factors play a crucial role in their efficacy against HCAs. The strong free radical scavenging activity of cumin extracts may be one of the key mechanisms underlying their inhibitory effects on *β*-carboline HCA formation. For practical application, determining the optimal range of cumin powder addition through experimentation is essential to maximize its inhibitory effect on HCA formation without unnecessarily increasing Harman content. Cumin from regions like Hetian should be prioritized due to its rich bioactive compounds and strong antioxidant capabilities, which can further enhance the safety and quality of smoked meat products. By leveraging these findings, cumin powder and its extracts can be more effectively integrated into food processing, particularly in the production of smoked meat, to reduce HCA formation and improve overall product safety and quality. Specifically, the use of cumin powder in food processing may involve the development of flavorful snack condiments, ready-to-eat meal kits, plant-based protein enhancers, artisanal bread mixes, and global fusion sauces containing cumin. These applications not only reduce the formation of HCAs, but also meet consumer needs for health, convenience and authenticity, adapting to evolving trends in the food industry.

While the current study provides valuable insights into the inhibitory effects of cumin extracts on the formation of *β*-carboline heterocyclic amines (HCAs) in smoked meat, it is not without limitations. Firstly, our analysis was confined to three specific origins of cumin. Additionally, the study was conducted under controlled laboratory conditions, which may not fully replicate the complexities of industrial-scale meat processing. For future research, it is recommended to expand the study to include a broader range of cumin varieties and to investigate the effects of cumin extracts on different food matrices. Longitudinal studies could also provide insights into the chronic effects of cumin consumption on HCA formation and human health. Moreover, exploring the synergistic effects of cumin with other natural inhibitors could lead to the development of more effective strategies to reduce HCA formation in food products. Finally, understanding the molecular mechanisms underlying the inhibitory effects of cumin extracts on HCA formation could open new avenues for targeted food fortification and health promotion.

## Figures and Tables

**Table 1:** *β*-Carbolines HAs-mixed aldehyde analogue system.

Reaction Precursor	Added Amount			
Glucos (mmol/mL)	0.02			
Creatinin (mmol/mL)	0.04			
Tryptoph (mmol/mL)	0.04			
Cumin ethanol Extract (mg/mL)	0	0.01	0.05	0.1

**Table 2:** ESR spectrometer parameters.

ESR Device Operation Parameters	Numerical Value
center field (G)	3360
sweep width (G)	100
sweep time (s)	30
sweep count (times)	3
microwave power (mW)	20
modulation amplitude (G)	1.0

**Table 3:** Total phenolic and total flavonoid contents of cumin extracts from different origins.

Origin	Total Phenolics (mg/g)	Total Flavonoids (mg/g)
Hami	1.49 ± 0.07 a	5.93 ± 0.18 b
Turpan	1.58 ± 0.04 a	6.25 ± 0.24 ab
Hetian	1.63 ± 0.07 a	6.7 ± 0.34 a

Data are presented as mean ± standard deviation (n = 3), with a significance level of *p* = 0.05. Different lowercase letters in the same column indicate significant differences (*p* < 0.05).

**Table 4:** The antioxidant capacity of cumin extracts from different origins.

Origin	ABTS (μM TE/g dw)	FRAP (μM TE/g dw)
Hami	79.32 ± 9.35 a	17.2 ± 0.51 b
Turpan	78.05 ± 2.52 a	19.76 ± 0.84 a
Hetian	85.12 ± 12.41 a	21.04 ± 1.22 a

Data are presented as mean ± standard deviation (n = 3), with a significance level of *p* = 0.05. Different lowercase letters in the same column indicate significant differences (*p* < 0.05).

## Data Availability

The original contributions presented in the study are included in the article, further inquiries can be directed to the corresponding authors.
